# Dickkopf-related protein 1, a new biomarker for local immune status and poor prognosis among patients with colorectal liver Oligometastases: a retrospective study

**DOI:** 10.1186/s12885-019-6399-1

**Published:** 2019-12-12

**Authors:** Qiaoqi Sui, Jian Zheng, Dingxin Liu, Jianhong Peng, Qingjian Ou, Jinghua Tang, Yuan Li, Lingheng Kong, Wu Jiang, Binyi Xiao, Xue Chao, Zhizhong Pan, Huizhong Zhang, Pei-Rong Ding

**Affiliations:** 1 Department of Colorectal Surgery, Sun Yat-sen University Cancer Center, Guangzhou, 510060 China; 2 State Key Laboratory of Oncology in South China, Guangzhou, 510060 China; 3 Collaborative Innovation Center for Cancer Medicine, Guangzhou, 510060 China; 4 Department of Experimental Research, Sun Yat-sen University Cancer Center, Guangzhou, 510060 China; 5 Department of Pathology, Sun Yat-sen University Cancer Center, Guangzhou, 510060 China

**Keywords:** Colorectal cancer, Liver oligometastases, DKK1, Immune status

## Abstract

**Background:**

It was reported that tumor-expressed dickkopf-related (DKK) proteins affect micro-environment. However, the influence of DKK1 on colorectal cancer (CRC) liver oligometastases (CRCLOM) remains unclear.

**Methods:**

CRC cases after resection of liver oligometastases were enrolled in Sun Yat-Sen University Cancer Center with intact clinical data. Serum DKK1 was detected by ELISA assay. Immunofluorescent staining examination for CD3 and CD8 in slices were also conducted.

**Results:**

Among 65 patients included, the recurrence-free survival (RFS) and overall survival (OS) were significantly better in the low serum DKK1 group (RFS: *P* = 0.021; OS: *P* = 0.043). DKK1 was overexpressed in stage IV CRC patients in TCGA data. The number of CD8+ tumor-infiltrating lymphocytes (TILs) in invasive margin of CRC liver oligometastases was significantly higher in low serum DKK1 group (*P* = 0.042).

**Conclusion:**

Elevated serum DKK1 level was associated with poorer RFS and OS, and less CD8+ TILs in invasive margin in CRC liver oligometastases. DKK1 might serve as a supplementalprognostic factor for clinical risk score and a potential target for immunotherapy.

## Background

Colorectal cancer (CRC) is the third most commonly diagnosed cancer globally [1]. About 35 to 50% of CRC will progress to distant metastasis, especially to the liver [2], and the recurrence risk and subsequent mortality of these patients after receiving traditional treatments remain very high [3]. Among CRC liver metastasis (CRCLM), CRC liver oligometastases (CRCLOM), with less than 5 lesions locating only in liver, is potentially curable and more likely to enjoy better survival after surgical treatments [4–6]. However, part of them are still suffering from early recurrence and poor prognosis according to existing studies [7]. Therefore, it is important to clinically identify CRCLOM patients who can benefit from liver resection. To date, clinical risk score (CRS) has been widely applied for evaluating the risk of recurrence after liver metastectomy [8], however, the accuracy remains controversial [9]. Recently, it was reported that the adaptive immune response in CRCLM may play a role in predicting the prognosis of CRCLOM patients [10–12], which indicates that biomarkers correlating to tumor immunity could be potential supplements for CRS.

As inhibitors of WNT signaling pathway, dickkopf-related (DKK) proteins were reported to suppress tumor progression [13, 14], while latest studies found that higher expression of DKK proteins were associated with worse prognosis as well as therapeutic resistance among different cancers [15–20]. Not just regulating its own signaling pathway in cancer cells [21], DKK proteins, especially DKK1, were also reported to affect microenvironments to influence tumor progression [16, 22, 23]. More significantly, Xiao revealed that DKK2 was highly expressed in *APC*-mutated CRC, inactivating CD8^+^ and NK cells and providing resistance to PD1 blockade [24].

However, the influence of DKK1 on CRCLM is unclear. Therefore, we conducted a study to explore the influences of DKK1 on CRCLOM and its impact on microenvironment.

## Methods

### Case inclusion and follow-up

This study was carried out in Sun Yat-sen University Cancer Center, and CRC cases diagnosed as CRCLOM who received metastasis resection between 2013 August 1st and 2016 December 31st were enrolled. The exclusion criteria are as follows: 1) With unavailable preoperative serum sample or metastatic lesions for slicing; 2) Developing CRCLOM after radical treatment of stage IV disease. Finally 65 patients were enrolled. Baseline information was collected. The CRS was calculated according to five risk factors: positive lymph nodes at the primary site, disease-free interval shorter than 12 months, number of metastases > 1, diameters of maximum metastases > 5 cm, and serum CEA levels > 200 ng/ml. Each risk factor was counted as one point if positive, corresponding to a possible score of 0–5 (8). As for follow-up, patients were monitored through subsequent visits every 3 months for the first 2 years and thereafter semiannually until 5 years after radical resection. The final follow-up visit occurred in July 2019. As comparison, 70 patients diagnosed stage I to III CRC who received surgery in our center were enrolled.

### DKK1 ELISA assay

Serum of 65 CRCLOM patients collected prior to liver resection were used for analysis, and serum collected prior to treatment of 70 stage I to III CRC cases between 2012 to 2016 were set as comparison. DKK1 levels were measured by human DKK1 ELISA Kit under the guidance of the manufacturer’s instructions (R&D Systems). For optimal measurements of DKK1 in the serum, samples were all 1:10 diluted with the dilution in the Kit. Based on ROC curve of recurrence, cut-off value of high and low DKK1 level was determined as the Youden index reached its maximum.

### Real-time quantitative PCR (qPCR)

Total RNA of CRCLOM frozen tissue was extracted with TRIzol (Invitrogen) and quantified on a ND-1000 spectrophotometer (NanoDrop Technologies). The cDNA was synthesized with 2 μg RNA using RevertAid First Strand cDNA Synthesis Kit (K1622, Thermo Scientific). The quantification of *DKK1* were determined using LightCycler 480 SYBR Green I Master (Roche) on LightCycler 480 Real-Time PCR System (Roche). The mRNA of *β-actin* was used as internal control. Relative quantification of transcription was calculated as the power of the difference between amplification of *DKK1* and amplification of *β-actin* (i.e., 2^-[Ct *DKK1* − Ct *β-actin*]^, where Ct represents threshold cycle).

Specific primers were as follows:

*DKK1*, 5′-CCTTGAACTCGGTTCTCAATTCC-3′.

and 5′-CAATGGTCTGGTACTTATTCCCG-3′;

*β-actin*, 5′-CAGGGCGTGATGGTGGGCATG-3′.

and 5′-GTAGAAGGTGTGGTGCCAGATT-3′.

### Correlation of *DKK1* expression and survival in TCGA

Data on *DKK1* expression was obtained from The Cancer Genome Atlas (TCGA) Pancancer Atlas data sets from cBioPortal (http://www.cbioportal.org/) [25, 26], and the overall survival (OS) data was obtained (https://www.cancer.gov/about-nci/organization/ccg/research/structural-genomics/tcga). Cut-off value of grouping was set based on ROC curve of OS.

### Immunofluorescent staining and immunohistochemistry (IHC) examination

Tissue sections of diagnosed colorectal cancer liver metastasis were used for lymphocyte counting. All specimens were prepared as 4 μm FFPE sections. Immunofluorescent staining was conducted according to Envision’s two steps manually in the light of the manufacturer’s instructions of DAKO. Paraffin-embedded slides of liver metastatic lesion specimens were stained using the primary monoclonal antibodies against CD3 (1:100, Abcam, Cambridge, UK) and CD8 (1100, Abcam, Cambridge, UK) proteins. The secondary was anti-rabbit/mouse IgG monoclonal antibody marked with fluorescence (DAKO Real Envision, Santa Clara, CA) in the dark, and DAPI was applied. Then, slices were covered with mounting medium (P36930, Invitrogen).

Immunohistochemical examination was performed to define center of the tumor (CT) and invasive margin (IM) area. Specimens were stained using an immunohistochemical technique that labeled the Keratin-positive (1:100, Abcam, Cambridge, UK) tumor cells, and definition of CT/IM was made by two pathologists (Additional file 2: Figure S1). Then, the CT/IM region in consecutive, immunofluorescent-stained slices were identified based on the distribution pattern of nucleus.

### Multispectral imaging

The stained slides were scanned using the Vectra System (Perkin Elmer), where one raw image comprising 3 stitched 200x multispectral image cubes for corresponding tissue areas. Each 200x multispectral captured the fluorescent spectra at 20 nm wavelength intervals from 420 to 720 nm with identical exposure time.

### Spectral unmixing and lymphocyte counting

For the sake of separating each multispectral image cube into its individual components (spectral unmixing) for the color-based identification of T-cell subtypes, the Nuance Imaging Analysis software (Perkin Elmer) was used to create spectral library containing the emitting spectral peaks of all fluorophores obtained from single stained slides for each marker and associated fluorophore. All spectrally unmixed and segmented images were then analyzed via inForm 2.1 image analysis software for counting. Based on the DAPI-stained nuclear, cell morphological features and patterns of fluorophore expression, cells were identified as CD3+ cells, CD8+ cells and CD3- other cells. CD3+ cells and CD8+ cells were counted in 5 area of IM and CT per slices randomly, and the average was calculated respectively. Two independent pathologists, who were blinded to the patients’ clinical information, participated in the analysis to verify the CT/IM region. Based on ROC curve of recurrence, cut-off value of high and low CD3 + CD8+ TIL was determined as the Youden index reached its highest value.

### Statistical analysis

SPSS 19.0 (Chicago, IL) and Prism 6 software (GraphPad) were used for data analysis. Data for continuous and discrete variables are reported as mean and median respectively. Data for categorized variables are reported as percentages. The Student’s *t* test was used for comparison of two sets of quantitative data. The Wald chi-square test was used to compare the difference of categorical parameters. Distributions of recurrence-free survival (RFS) and overall survival (OS) were described by Kaplan-Meier methods. Univariate and multivariate Cox proportional hazards models were used to predict outcome influential factors. Receiver operating characteristic (ROC) curves were also used to compare the predictive ability of the prognostic factors for survival. All *P* values were two-sided, with *P*<0.05 as statistically significant.

## Results

### Patient characteristics

**Fig. 1:**
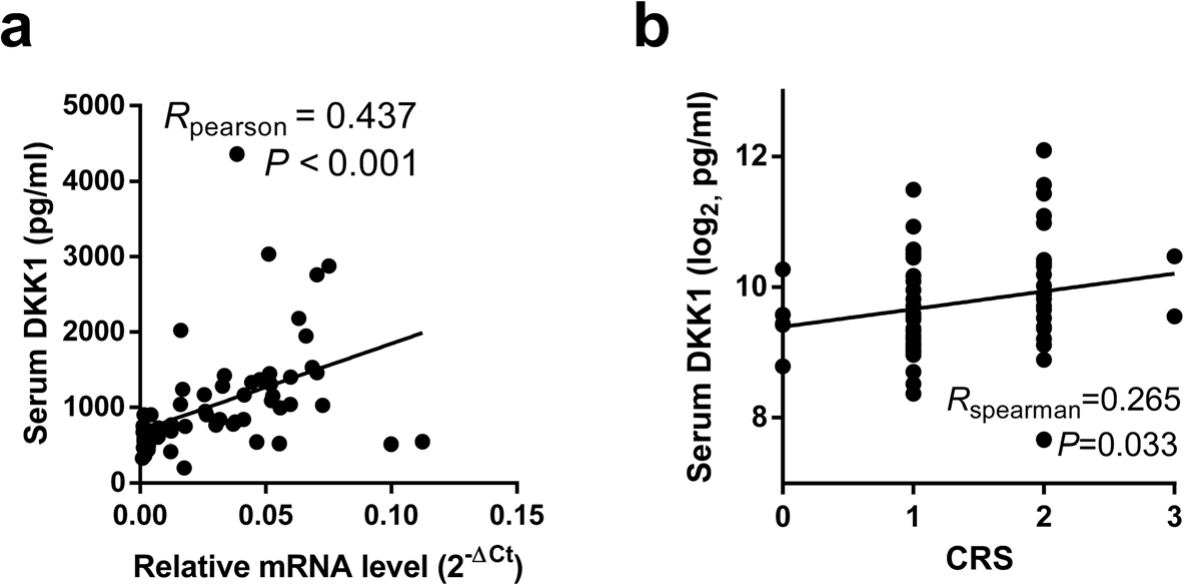
**a** Pearson correlation test of the correlation between serum DKK1 level and relative *DKK1* mRNA level in metastases lesions. **b** Spearman rank correlation test of the correlation between serum DKK1 level and CRS scores

Sixty-five patients with CRCLOM were included. ELISA assay was conducted for serum DKK1 level, and qPCR was done for quantification of *DKK1* mRNA expression in metastatic lesions. Pearson correlation test demostracted that serum DKK1 level positively correlated to *DKK1* expression in metastatic lesion (R = 0.437, *P* < 0.001, Fig. 1a). The mean serum DKK1 level was 1037.7 pg/ml. Of the 65 patients, 28 (43.1%) had low serum DKK1, whereas 37 (56.9%) had high DKK1. The cut-off value was 747 pg/ml, which reach the optimal diagnostic efficiency according to Youden index (Table 1).

**Table 1:** Baseline characteristics of mCRC cases

	DKK1 low serum level (%)	DKK1 high serum level (%)	*P*
Total	28	37	
Gender			0.683
Male	16 (57.1)	23 (62.2)	
Female	12 (42.9)	14 (37.8)	
Median age, range	56, 32~75	59, 30~76	0.454
Splenic flexure (primary tumor)			0.271
Proximal	5 (17.9)	11 (29.7)	
Distal	23 (82.1)	26 (70.3)	
Pathological determination			0.211
Adenocarcinoma, NOS	28 (100.0)	35 (94.6)	
Mucous adenocarcinoma	0 (0.0)	2 (5.4)	
R0 resection			0.278
Yes	27 (96.4)	33 (89.2)	
No	1 (3.6)	4 (10.8)	
Interventional therapy			0.773
Yes	26 (92.9)	35 (94.6)	
No	2 (7.1)	2 (5.4)	
Preoperative chemotherapy			0.190
Yes	7 (25.0)	15 (40.5)	
No	21 (75.0)	22 (59.5)	
Regimen of preoperative chemotherapy			
XELOX	2	8	
FOLFOX	2	5	
FOLFIRI	3	2	
Postoperative chemotherapy			0.046
Yes	12 (42.9)	25 (67.6)	
No	16 (57.1)	12 (32.4)	
Regimen of postoperative chemotherapy			
XELODA	0	1	
XELOX	9	11	
FOLFOX	1	7	
FOLFIRI	1	4	
XELOX+XELODA	1	2	
Preoperative CEA level (ng/L)			0.211
≤ 200	28 (100.0)	35 (94.6)	
> 200	0 (0.0)	2 (5.4)	
Primary tumor node			0.058
Positive	10 (35.7)	22 (59.5)	
Negative	18 (64.3)	15 (40.5)	
Disease-free interval			0.161
< 12 months	25 (89.3)	28 (75.7)	
≥ 12 months	3 (10.7)	9 (24.3)	
Tumor size			0.043
≤ 5 cm	28 (100.0)	32 (86.5)	
> 5 cm	0 (0.0)	5 (13.5)	
CRS			0.347
0	3 (10.7)	2 (5.4)	
1	15 (53.6)	15 (40.5)	
2	10 (35.7)	18 (48.6)	
3	0 (0)	2 (5.4)	

^a^ mCRC, metastatic colorectal cancer; NOS, not otherwise specified; CRS, clinical risk score

Patients with higher serum DKK1 were associated with larger-sized (diameters> 5 cm) metastasis (*P* = 0.043, Table 1). There was a tendency that serum DKK1 is associated with lymph node metastasis (*P* = 0.058, Table 1). Spearman rank correlation test further revealed that serum DKK1 level positively correlated to CRS scores (R = 0.265, *P* = 0.033, Fig. 1b). The baseline information of the patients is presented in Table 1.

### Prognostic value of serum DKK1 in CRCLOM

**Fig. 2:**
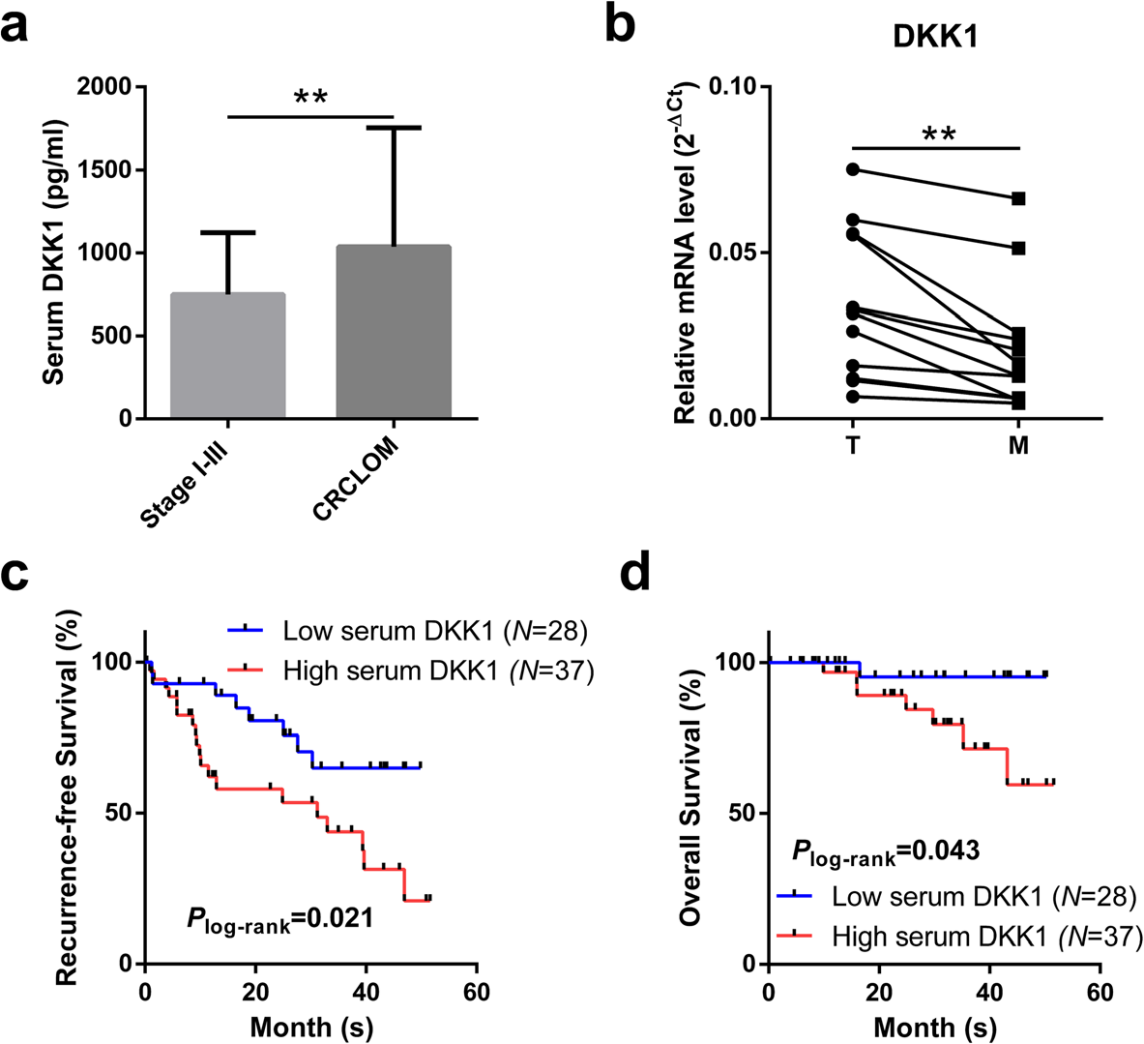
**a** Comparison of preoperative serum DKK1 level between stage I to III CRC and CRCLOM patients. **b** Comparison of relative *DKK1* expression between 12 pairs of primary and metastatic leisions. T: primary tumors; M: metastatic lesions. **c** and **d** Kaplan–Meier curves comparing recurrence-free survival **c** and overall survival **d** of CRC liver oligometastases patients with high and low serum DKK1. **: 0.001 ≤ *P* < 0.01

Compared with stage I~III CRC patients, preoperative serum DKK1 level was significantly higher in CRCLOM patients (*P* = 0.004, Fig. 2a). Besides, in 12 CRCLOM cases with paired primary tumor, *DKK1* expression was significantly higher in metastastic lesions. (*P* = 0.002, Fig. 2b). In addition, elevated serum DKK1 was associated with risk of recurrence (*P* = 0.021, Fig. 2c) and poor overall survival (*P* = 0.043, Fig. 2d), while multivariate Cox proportional hazards regression analysis demostrated that DKK1 independently correlated to neither recurrence (*P* = 0.150, Table 2) nor overall survival (*P* = 0.122, Additional file 1: Table S1).

**Table 2:** Univariate and multivariate Cox proportional hazards regression models for RFS

Variate	No. of case	*HR* (95% *CI*)	*P*
DKK1 serum level			
Low	28	1.000 (ref)	–
High	37	2.565 (1.117~5.888)	0.026
Primary tumor node			
Negative	33	1.000 (ref)	–
Positive	32	2.002 (0.920~4.358)	0.080
Disease-free interval			
< 12 months	53	1.000 (ref)	–
≥ 12 months	12	0.618 (0.450~3.829)	0.618
Preoperative CEA level (ng/L)			
≤ 200	63	1.000 (ref)	–
> 200	2	4.415 (0.574~33.988)	0.154
Tumor size			
≤ 5 cm	60	1.000 (ref)	–
> 5 cm	5	3.944 (1.331~11.688)	0.013
Multivariate			
DKK1 serum level			
Low		1.000 (ref)	–
High		1.943 (0.786~4.803)	0.150
Tumor size			
≤ 5 cm		1.000 (ref)	–
> 5 cm		2.713 (0.883~8.331)	0.262
Primary tumor node			
Negative		1.000 (ref)	–
Positive		1.519 (0.672~3.432)	0.315

### Association between DKK1 expression and prognosis among stage IV CRC in TCGA database

**Fig. 3:**
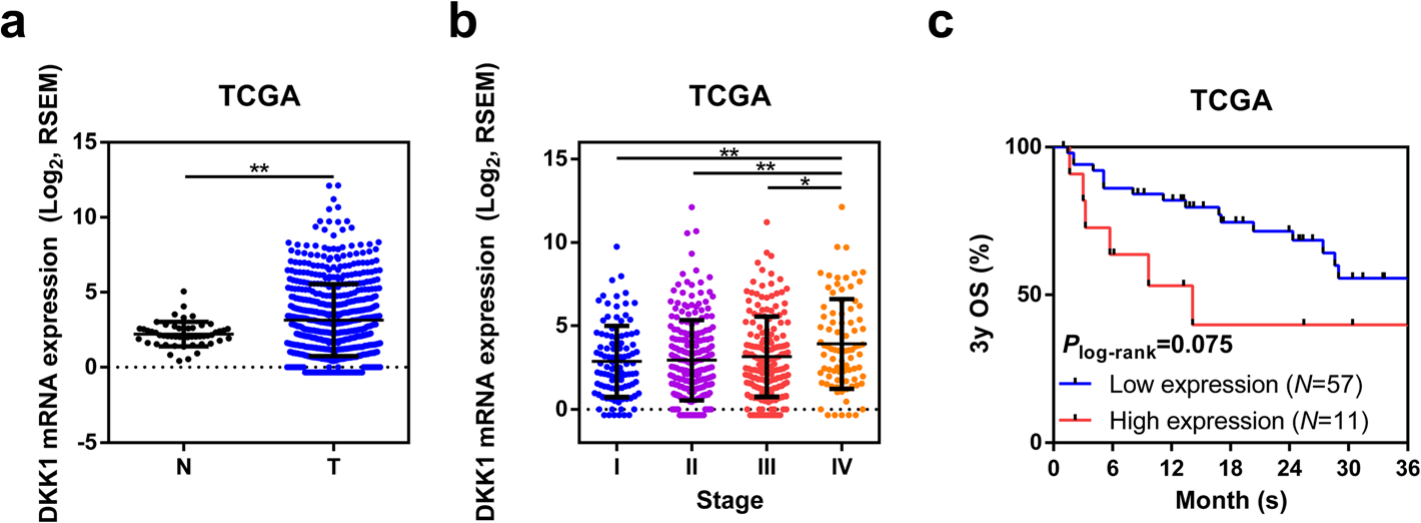
**a** Analysis of the expression of *DKK1* in normal colorectal tissues and CRCs in TCGA data sets. **b** Analysis of the expression of *DKK1* in different stages of CRCs. **c** Kaplan–Meier curves comparing 3 year overall survival (3y OS) of stage IV patients with high and low *DKK1* expression. *: 0.01 ≤ *P* < 0.05; **: 0.001 ≤ *P* < 0.01

Analysis of The Cancer Genome Atlas Network (TCGA) data sets from cBioPortal further revealed that the expression of DKK1 is significantly different between normal colorectal tissues and CRCs (Fig. 3a). Furthermore, *DKK1* expressions is significantly higher in stage IV tumors (Fig. 3b). Among stage IV patients, 3-year overall survival (3y OS) showed a tendency that high DKK1 expression correlates with poor survival rates (*P* = 0.075, Fig. 3c).

### Association between serum DKK1 and tumor-infiltrating lymphocytes (TILs) in CT and IM

**Fig. 4:**
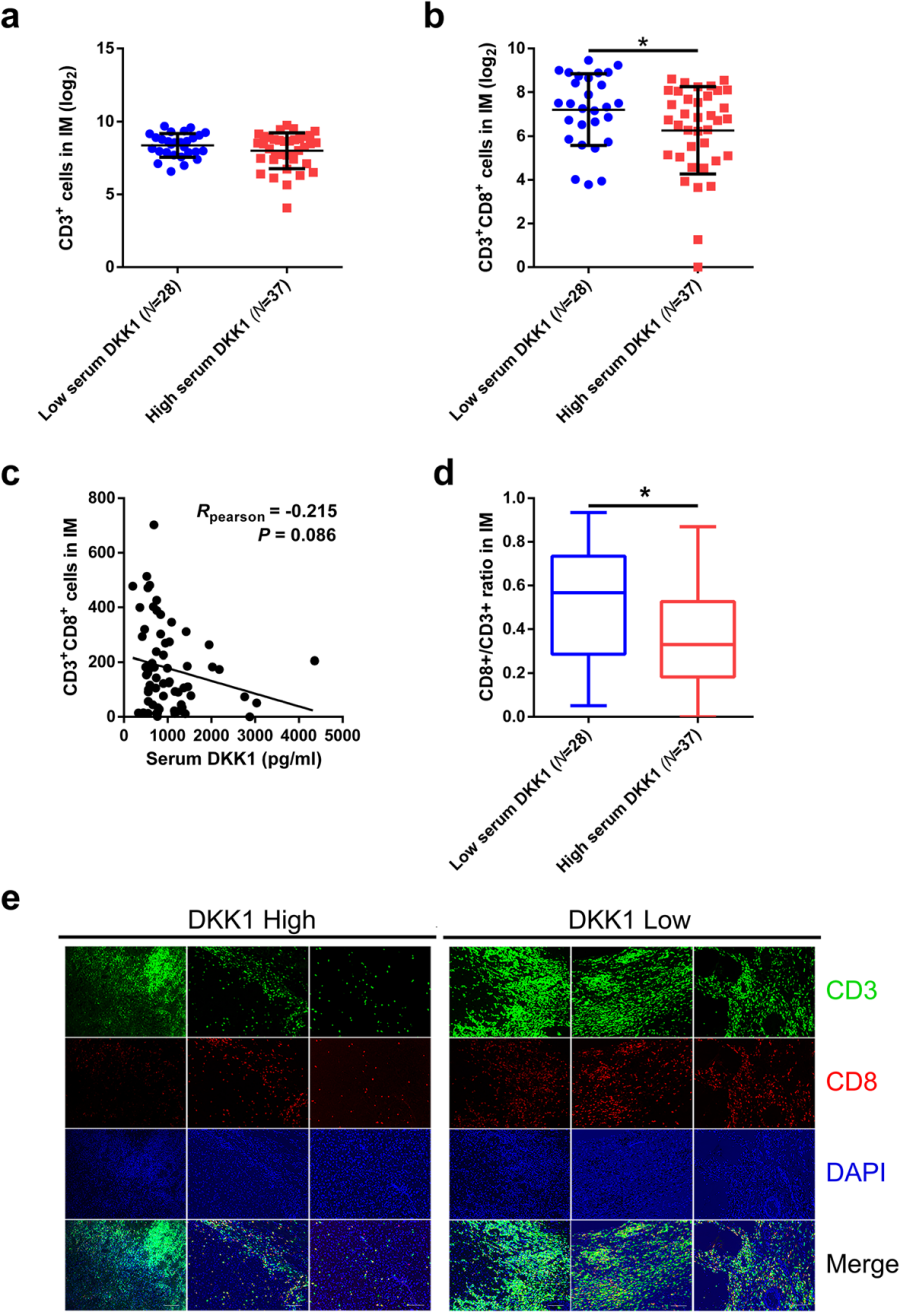
**a** and **b** The numbers of total CD3+ TILs **a** and CD3 + CD8+ TILs **b** in invasive margin (IM) with low and high serum DKK1. **c** Pearson correlation test of the correlation between serum DKK1 level and the number of CD3 + CD8+ T cells in IM. **d** Comparison of CD8+/CD3+ ratio in IM between two groups of CRCLOM. **e** Typical image of immunofluorescence staining of metastasis slices. Scale bars represent 100 μm. *: 0.01 ≤ *P* < 0.05

In IM, the numbers of total CD3+ TILs were not significantly different between two groups, while CD3 + CD8+ T cells were significantly higher in low serum DKK1 group (*P* = 0.042, Fig. 4a). Also, The Pearson rank correlation test showed that numbers of CD3+ CD8+ T cells in IM trended to negatively correlated to DKK1 serum level (*R* = -0.215, *P* = 0.086, Fig. 4b). In addition, the CD8+/CD3+ ratios in IM was higher in low serum DKK1 group (*P* = 0.015; Fig. 4c). Typical image of immunofluorescence staining of metastasis slices were shown (Fig. 4d). Furthermore, Elevated CD3 + CD8+ cells in IM correlated to better RFS (*P* = 0.026; Additional file 3: Figure S2A), while not significance was detected in OS (*P* = 0.810; Additional file 3: Figure S2B). The numbers of total CD3+, CD3 + CD8+ TILs and CD8+/CD3+ ratio in CT were not significantly different between two groups (Additional file 3: Figure S2C-E).

ROC curves were used to compare the sensitivity and specificity of RFS prediction between serum DKK1 level and CD3 + CD8+ cell counting in IM. In the ROC analysis, the CD3 + CD8+ cell counting had significant predictive values, with an area under the ROC curve (AUC) of 0.646 (95% *CI* 0.508–0.785, *P* = 0.046, Additional file 4: Figure S3), while the predictive value of serum DKK1 level was not significant, with an AUC of 0.615 (95% *CI* 0.476–0.754, *P* = 0.116). Although the CD3 + CD8+ cell counting corresponded to a larger AUC for RFS prediction, the difference was not statistically significant (*P* = 0.757).

## Discussion

In the present study, the ELISA examination was first applied to evaluate serum DKK1 in CRCLOM patients, showing a prognostic value both efficient and convenient. More significantly, CD3 + CD8+ TIL accumulation and CD8+/CD3+ ratio in metastasis IM was worse with elevated serum DKK1.

Serum DKK1 level positively correlated to *DKK1* expressions in CRCLOM lesions. Based on D’Amico, serum DKK1, although not always tumor-expressed, was associated with tumor expression in pancreatic cancer model [22]. In addition, Zhuang reported that the *DKK1* expression correlates to tumor-secreted DKK1 in breast cancer [23]. Together with those findings, screening of serum DKK1 could be appropriate for reflecting tumor-expressed DKK1.

Elevated serum DKK1 were reported to be associated with poor prognosis among various tumors [15–18], whereas *DKK1* was also reported to be a tumor suppressor gene conventionally [27]. While in the current study, DKK1 was associated with liver metastasis and risk of recurrence in CRC. Furthemore, elevated DKK1 correlated to worse prognosis and adverse clinical features, especially larger metastasis size. Additionally, analysis of TCGA database revealed that expression of *DKK1* was elevated in M1 stage compared to M0 stage CRC tissue, and trended to correlate to poor prognosis among metastatic disease. Therefore, our study indicated that DKK1 correlates to progression and poor prognosis in CRCLOM.

In the current study, serum DKK1 served to be a prognostic predictor, associated with distant metastasis, larger metastasis size and higher CRS, and we believed higher CRS could explain why patients were more likely to receive postoperative chemotherapy with higher DKK1. Prior to our study, researchers have pointed out that DKK1 correlates to clinical features affecting prognosis among a variety of tumors. Shi reported that tumor-expressed DKK1 is an independent prognostic factor for both RFS and OS, correlating to metastasis of hepatic hilar lymph nodes among intrahepatic cholangiocarcinoma (ICC) [28]. In addition, Liu advocated that DKK1 is an independent predictor of overall survival in patients with pancreatic ductal adenocarcinoma (PDAC), associated with lymph node metastasis and T stage classification as well [29]. Since the inaccuracy of CRS system was revealed when predicting prognosis [9], we considered serum DKK1 test could serve as a compliment on recurrence prediction.

Despite inhibiting Wnt-signaling pathway in cancer cells, DKK proteins were found promoting tumor progression via several mechanisms [20, 21, 30]. In the current study, we analyzed the correlation between serum DKK1 and TILs in CRCLOM. Immunofluorescent staining examination revealed that DKK1 correlated to the accumulation of CD3 + CD8+ TIL and CD8+/CD3+ ratio in IM negatively. According to D’Amico, DKK1 activates Myeloid-derived suppressor cell (MDSC), a suppressor of tumor immunology, to indirectly reduce accumulation of CD4+ and CD8+ TILs in Lewis lung carcinoma, pancreatic cancer as well as B16 melanoma [22]. Also, MDSC is well-known to be a modulator of regulatory T cells (Treg cells) [31], which could contribute to the dismiss of CD8+ T cells and lower CD8+/CD3+ ratio. Furthermore, DKK2, another DKK protein, was reported directly inhibiting CD8+ T cells in APC mutated CRC models via LRP5 [24], a receptor which could also combine DKK1 [32]. Here, our study indicated that DKK1 could also worsen immune status in colorectal cancer.

Mlecnik revealed that the adaptive immune response in mCRC, including accumulation of CD3+ and CD8+ immune cells, may play a role in preventing tumor recurrence and poor overall survival [33]. Subsequently, Katz SC reported that higher CD8+ T cell counting in CRC liver metastasis was associated with higher 10-year survival rate after metastasis resection [34]. Also, he demonstrated that CD8+/CD3+ ratio predicted better prognosis [35]. However, existing studies suggested that prognosis in CRC liver metastasis is governed by the state of the local adaptive immune response in both CT and IM [9, 33, 36], while DKK1 serum level negatively correlates to CD8+ TIL accumulation in IM, but not CT in the current study. IM is the primary site of interaction between malignant cells and immune cells in metastasis [10], thus supposed to be the location where DKK1 takes effect. Halama reported that density of CD3+, CD8+ TILs and granzyme-B+, FOXP3+ immune cells at the IM of CRCLM predicts prognosis and sensitivity to chemotherapy [10], which supports our hypothesis that DKK1 cause poor prognosis via dismiss CD8+ TILs in IM. Apart from that, with overexpression of intrinsic immunosuppressive oncogenic pathways and low expression of tumor-specific antigen [37], a single factor may be less likely to influence the immunosuppressive status in CT.

It has been revealed that microsatellite instability (MSI) among CRC patients enjoy better prognosis, low risk of liver metastasis and sensitivity to immune checkpoint blockade due to fine immune status [38–41]. Now that elevated serum DKK1 was associated with dismissed CD8 + TILs, targeting DKK1 may improve the immune status in CRCLOM. It was not a new idea that DKK proteins may be promising targets for therapy against cancer disease, as anti-DKK1 had been reported beneficial for multiple myeloma [42].

There are certain limitations in the current study. First of all, it was a retrospective study containing a small sample of only 65 patients, which could lead to bias during multivariate Cox proportional hazards regression analysis. Secondly, we lacked postoperative serum, thus unable to explore the change of serum DKK1 after surgery. Last but not least, mechanism of the affection on CD8+ TILs by DKK1 were not clarified, and No clinical data proves that targeting DKK1 contributes to stronger immune response.

In conclusion, the current study demonstrates that elevated serum DKK1 level was associated with worse recurrence-free survival and poor CD8+ TILs in IM in CRCLOM. The findings might serve as supplement for clinical risk score and a potential target for immunotherapy.

## Supplementary information

**Additional file 1:** **Table S1.** Univariate and multivariate Cox proportional hazards regression models for OS.

**Additional file 2:** **Figure S1.** Typical image of CT/IM definition using IHC examination.

**Additional file 3:** **Figure S2.** (A and B) Kaplan–Meier curves comparing recurrence-free survival (A) and overall survival (B) of CRCLOM patients with high and low CD3 + CD8+ TIL. The numbers of total CD3+ TILs and CD3 + CD8+ T cells in center of the tumor (CT) with low and high serum DKK1. (C and D) The numbers of total CD3+ TILs (C) and CD3 + CD8+ TILs (D) in CT with low and high serum DKK1. (E) Comparison of CD8+/CD3+ ratio in CT between two groups of CRCLOM.

**Additional file 4:** **Figure S3.** Comparison of the sensitivity and specificity for predicting RFS of CRCLOM patients with serum DKK1 level and number of CD8 + TIL in IM.

## Data Availability

The datasets used and/or analysed during the current study are available from the corresponding author on reasonable request.

## Abbreviations

3y OS: 3-year overall survival
CRC: Colorectal cancer
CRCLM: Colorectal liver metastasis
CRCLOM: Colorectal liver oligometastasis
CRS: Clinical risk score
CT: Center of the tumor
DKK: Dickkopf-related
ICC: Cholangiocarcinoma
IHC: Immunohistochemistry
IM: Invasive margin
mCRC: Metastatic colorectal cancer
MDSC: Myeloid-derived suppressor cell
MSI: Microsatellite instability
OS: Overall survival
PDAC: Pancreatic ductal adenocarcinoma
RFS: Recurrence-free survival
TCGA: The Cancer Genome Atlas
TILs: Tumor-infiltrating lymphocytes
Treg cells: Regulatory T cells
