# Supplementary material for: Dickkopf-related protein 1, a new biomarker for local immune status and poor prognosis among patients with colorectal liver Oligometastases: a retrospective study
Source: BMC Cancer. 2019 Dec 12;19:1210. doi: 10.1186/s12885-019-6399-1 (PMC6909492; doi:10.1186/s12885-019-6399-1)
Supplement: Supplementary file 1 — Additional file 1: Table S1. Univariate and multivariate Cox proportional hazards regression models for OS. [file 12885_2019_6399_MOESM1_ESM.docx]

Supplementary Table. Univariate and multivariate Cox proportional hazards regression models for OS.

| **Variate** | **No. of case** | ***HR* (95% *CI*)** | ***P*** |
| --- | --- | --- | --- |
| **DKK1 serum level** |  |  |  |
| Low | 28 | 1.000 (ref) | —— |
| High | 37 | 6.559 (0.806~53.404) | 0.079 |
| **Primary tumor node** |  |  |  |
| Negative | 33 | 1.000 (ref) | —— |
| Positive | 32 | 2.044 (0.487~8.587) | 0.329 |
| **Tumor size** |  |  |  |
| ≤5cm | 60 | 1.000 (ref) | —— |
| >5cm | 5 | 4.349 (0.871~21.712) | 0.073 |
| **Multivariate** |  |  |  |
| **DKK1 serum level** |  |  |  |
| Low |  | 1.000 (ref) | —— |
| High |  | 2.503 (0.482~12.998) | 0.122 |
| **Tumor size** |  |  |  |
| ≤5cm |  | 1.000 (ref) | —— |
| >5cm |  | 2.713 (0.883~8.331) | 0.262 |
|  |  |  |  |
