# Supplementary figures and images for: Dickkopf-related protein 1, a new biomarker for local immune status and poor prognosis among patients with colorectal liver Oligometastases: a retrospective study
Source: BMC Cancer. 2019 Dec 12;19:1210. doi: 10.1186/s12885-019-6399-1 (PMC6909492; doi:10.1186/s12885-019-6399-1)

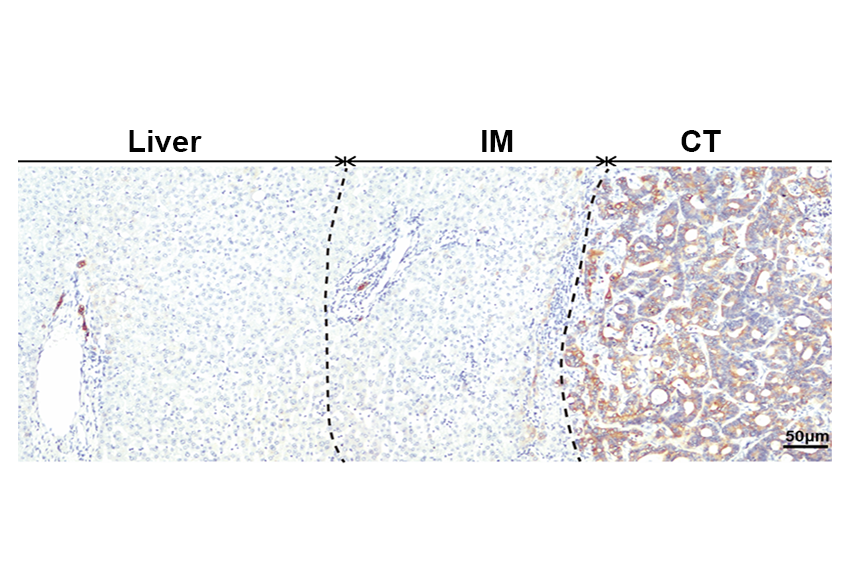

Supplement: Supplementary file 2 — Additional file 2: Figure S1. Typical image of CT/IM definition using IHC examination. [file 12885_2019_6399_MOESM2_ESM.tif]

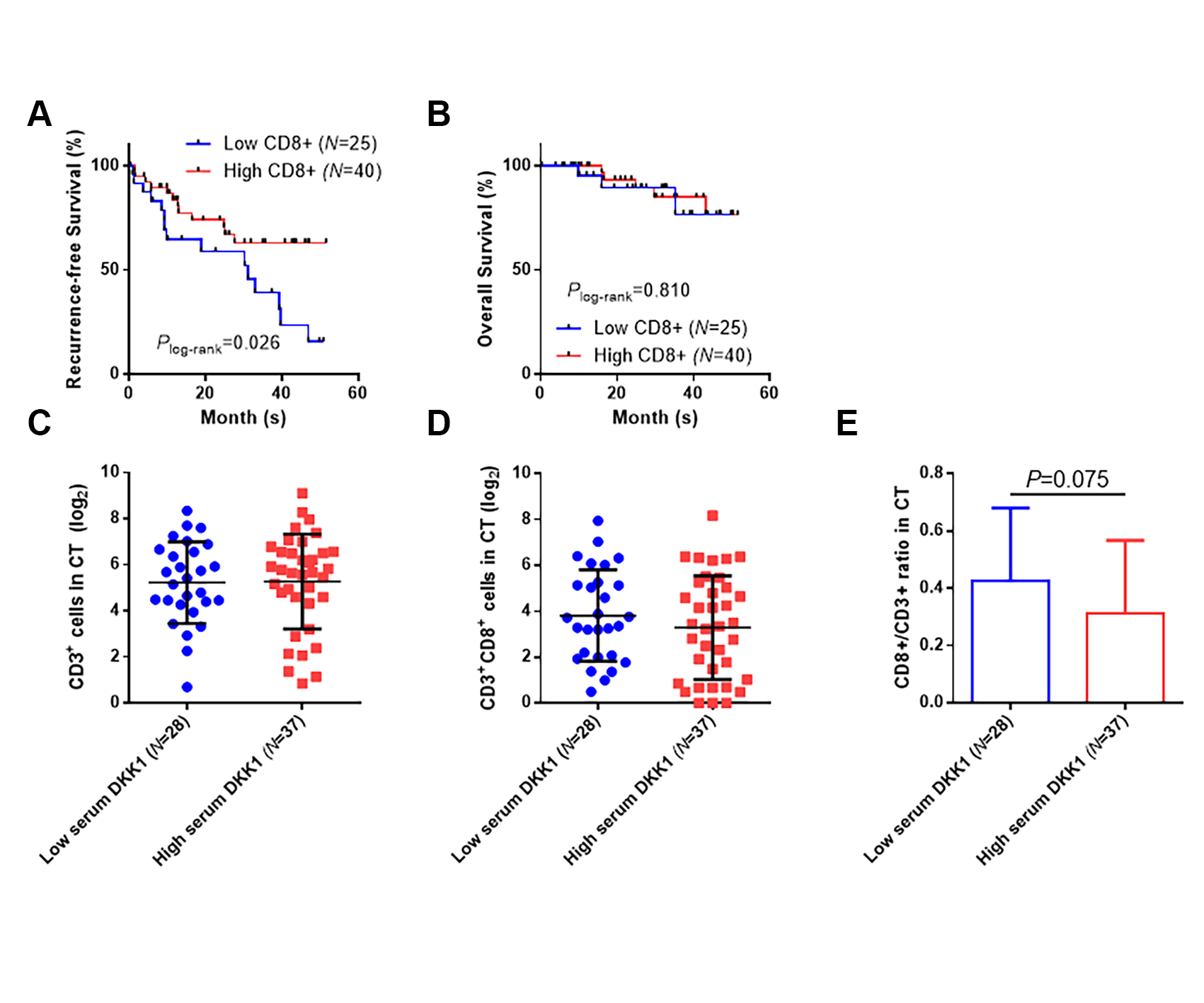

Supplement: Supplementary file 3 — Additional file 3: Figure S2. (A and B) Kaplan–Meier curves comparing recurrence-free survival (A) and overall survival (B) of CRCLOM patients with high and low CD3 + CD8+ TIL. The numbers of total CD3+ TILs and CD3 + CD8+ T cells in center of the tumor (CT) with low and high serum DKK1. (C and D) The numbers of total CD3+ TILs (C) and CD3 + CD8+ TILs (D) in CT with low and high serum DKK1. (E) Comparison of CD8+/CD3+ ratio in CT between two groups of CRCLOM. [file 12885_2019_6399_MOESM3_ESM.tif]

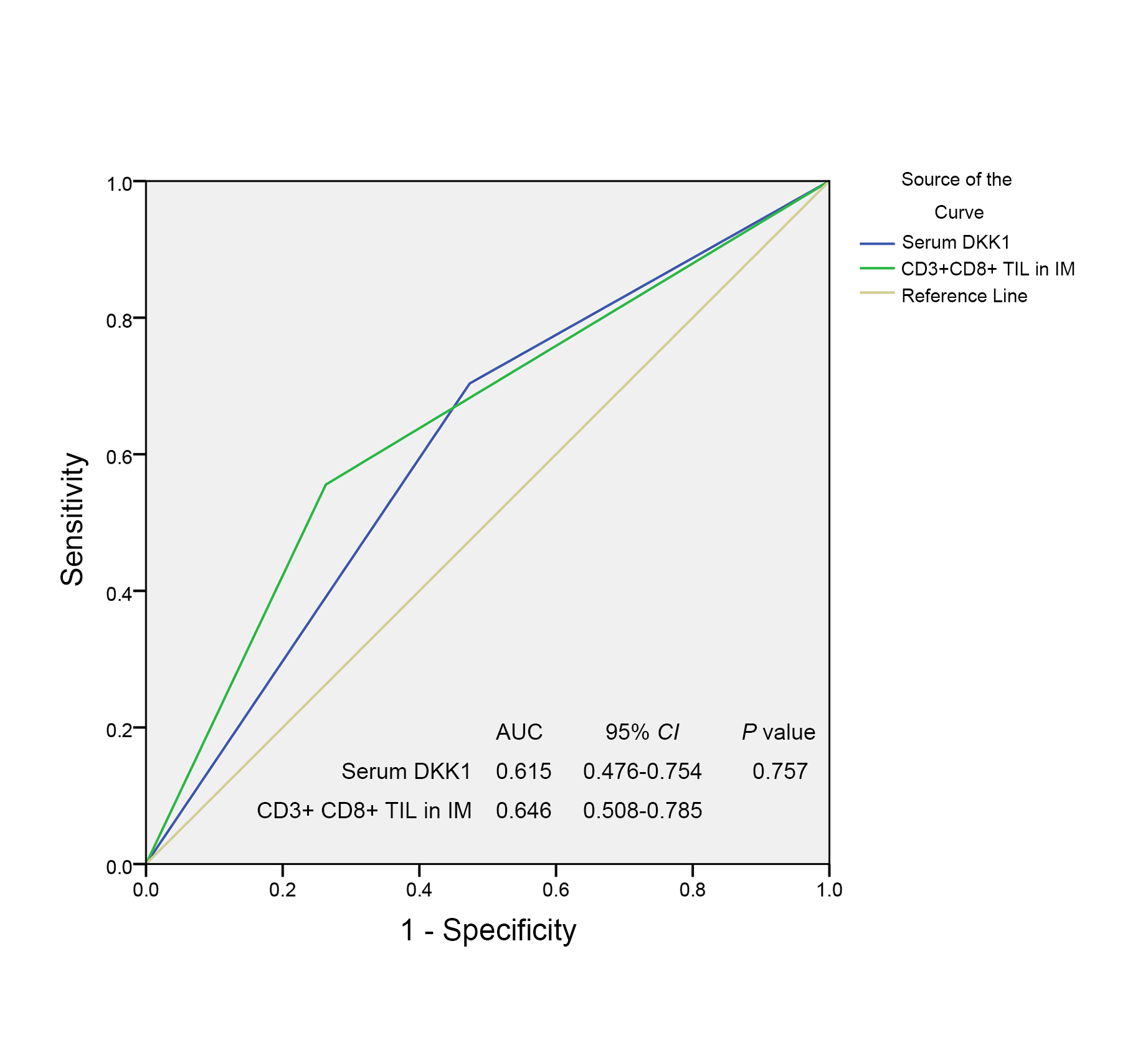

Supplement: Supplementary file 4 — Additional file 4: Figure S3. Comparison of the sensitivity and specificity for predicting RFS of CRCLOM patients with serum DKK1 level and number of CD8 + TIL in IM. [file 12885_2019_6399_MOESM4_ESM.tif]
